# Mechanically Tunable Poly(Ethylene Glycol) Diacrylate Hydrogels Reveal Stiffness‐Related Impairments in Capillary Sprouting in Experimental Lung Fibrosis

**DOI:** 10.1111/micc.70018

**Published:** 2025-07-17

**Authors:** Julie Leonard‐Duke, Samuel M. J. Agro, David J. Csordas, Riley T. Hannan, Anthony C. Bruce, Jeffrey M. Sturek, Shayn M. Peirce, Lakeshia J. Taite

**Affiliations:** ^1^ Department of Biomedical Engineering University of Virginia Charlottesville Virginia USA; ^2^ Robert M. Berne Cardiovascular Research Center University of Virginia Charlottesville Virginia USA; ^3^ Department of Chemical Engineering University of Virginia Charlottesville Virginia USA; ^4^ Department of Pulmonary and Critical Care Medicine University of Virginia Charlottesville Virginia USA

**Keywords:** angiogenesis, fibrosis, hydrogels, poly(ethylene glycol)

## Abstract

**Objective:**

Synthetic hydrogels that support 3D cell culture are widely used as platforms for modeling disease, such as tissue fibrosis, which leads to mechanical stiffening of the extracellular matrix (ECM). To interrogate how mechanical stiffness of the ECM affects microvascular remodeling, we developed a bioactive poly(ethylene glycol) diacrylate (PEGDA) hydrogel model with tunable stiffness that permits microvascular sprouting.

**Methods:**

Lung explants harvested from healthy and fibrotic mice were cultured ex vivo on PEGDA hydrogels for 7 days. Capillary sprouting from lung segments was evaluated via imaging and secreted angiogenic markers.

**Results:**

Healthy lung explants had decreased sprout formation and length on stiffer hydrogels. The sprouts from fibrotic lung explants, however, were not impacted by hydrogel stiffness. This difference was associated with higher expression of angiogenic markers and matrix remodeling enzymes in the fibrotic lung explants.

**Conclusions:**

Our results suggest a compensation in vasculature derived from fibrotic tissue to matrix mechanics in promoting angiogenic sprouting.

## 1 Introduction

Fibrotic disease is characterized by progressive stiffening of the extracellular environment, and previously published studies have reported that the mechanical stiffness of the extracellular environment affects angiogenic sprouting [1]. Therefore, it is important to understand how the mechanical properties of the extracellular environment impact angiogenic sprouting of capillaries residing in soft healthy tissues compared to stiff fibrotic tissues. Lung fibrosis is a particularly interesting fibrotic disease context in which to study the impacts of extracellular environmental stiffness on angiogenic sprouting because both increased and decreased capillary sprouting have been observed; and both contribute to lung function decline [2, 3, 4]. It is difficult to study this microvascular sprouting and pruning in in vivo models of lung fibrosis primarily because the lungs are encased in the ribs which prevents the penetration of non‐invasive imaging techniques and requires invasive surgery to remove the ribs which not only poses additional risk but limits the length of the study as the mouse has to be continuously monitored and euthanized at the end of image acquisition [5]. Additionally, the constant movement of the lungs and high air content can make in vivo imaging difficult. This necessitates the development of in vitro and ex vivo models to study vascular remodeling.

Poly(ethylene glycol) diacrylate (PEGDA) hydrogels can be used as a substrate for modeling angiogenic sprouting in healthy and fibrotic microenvironments because their mechanical properties can be adjusted through various means, such as crosslinking or functionalization of polymer end groups [6, 7, 8, 9, 10, 11]. The versatile diacrylate crosslinking mechanism allows for further functionalization of the matrix, such as with acrylate‐functionalized peptides or other biomolecules [1, 9, 10, 12, 13, 14, 15]. This property confers bioactivity to the inert poly(ethylene glycol) (PEG) scaffold, such as the incorporation of pro‐angiogenic growth factors like vascular endothelial growth factor A (VEGF), platelet‐derived growth factor BB (PDGF), and fibroblast growth factor 2 (FGF) [16, 17, 18, 19]. Furthermore, the incorporation of protease‐sensitive domains into the polymer backbone permits cell‐mediated degradation and multicellular assembly [12, 13, 14]. Strict control over hydrogel properties facilitates the design of disease models that can isolate the impact of mechanical stiffness on cell behavior independent of biochemical factors. In our prior work, we used PEGDA hydrogels to create a novel ex vivo lung explant assay to study how different levels of environmental stiffness impacted vascular sprouting in healthy lung [1]. We found that higher hydrogel stiffnesses, representing the stiffness of lung fibrotic lesions [20], significantly reduced capillary sprouting from healthy lung capillaries [1]. This was found to be driven by the mechanical properties of the gel and independent of pro‐angiogenic growth factor treatment.

Based on reports of decreased capillary density in fibrotic foci in IPF [2, 3, 4] and our previous results [1], in the present work, we test the hypothesis that capillaries residing in fibrotic lung tissue will exhibit impaired capillary sprouting relative to capillaries residing in healthy lung, and that this sprouting impairment will be exacerbated in a stiffened extracellular environment. To do this, we leveraged the murine bleomycin model of lung fibrosis, which is well‐characterized and widely used by our groups and others [21, 22, 23, 24, 25, 26]. Bleomycin is a chemotherapeutic agent that induces lung inflammation and fibrotic lesion formation in murine lungs over the course of 14–28 days [24, 25]. By harvesting lung explants from both healthy and fibrotic mice and embedding them in PEGDA hydrogels of varying stiffnesses to represent healthy and diseased microenvironments, we determined how mechanical stiffness of the extracellular environment differentially affects angiogenic sprouting from healthy and diseased capillaries. Additionally, we quantified the secretion of pro‐angiogenic cytokines and matrix metalloproteases (MMPs) by the healthy and fibrotic lung explants in both soft and stiff microenvironments to ascertain the soluble, secreted molecular drivers of angiogenic sprouting.

## 2 Methods

### 2.1 Mice and Bleomycin Administration

All animal procedures were performed with the approval and in accordance with the Animal Care and Use Committee guidelines at the University of Virginia. Eight‐week‐old C57BL/6J male mice were purchased from Jackson Laboratories. To perform the bleomycin administration, each mouse was first anesthetized with ketamine/xylazine (Covetrus). A single dose of 1–3 units/kg of pharmaceutical grade bleomycin (Cardinal Health) in 50 μL sterile PBS was applied to the posterior oropharynx and aspirated into the lungs. Mice were then housed for 28 days prior to sacrifice. Seven mice in total were included in the study; four of the seven received bleomycin.

### 2.2 Lung Harvest and Explant Preparation

Four weeks after bleomycin administration, mice were euthanized by carbon dioxide asphyxiation followed by cervical dislocation to confirm death. Next, a cardiac perfusion was performed with 10 mL phosphate buffered saline (PBS). The lungs were isolated from the heart and other connective tissue, then placed into individual baths of cold PBS. To prepare the individual explants, the left lobe was separated from the right lobes and placed on a tissue slicer with the apical side against the blade. The lung was cut into 1 mm thick strips, which were then rotated and sliced in 1 mm increments again to produce 1 mm by 1 mm explants. In order to ensure that all regions of the lung were accounted for in each group to mitigate the effects of variable bleomycin delivery, each explant was placed into a well of a 96‐well plate containing cold PBS based on its location in the lobe. In total, approximately 25–30 samples were collected from the left lobe of each set of lungs. Of these 30 samples, 12 were used in each plate, six per hydrogel stiffness group.

### 2.3 Confirmation of Fibrotic Phenotype After Bleomycin Administration

**FIGURE 1:**
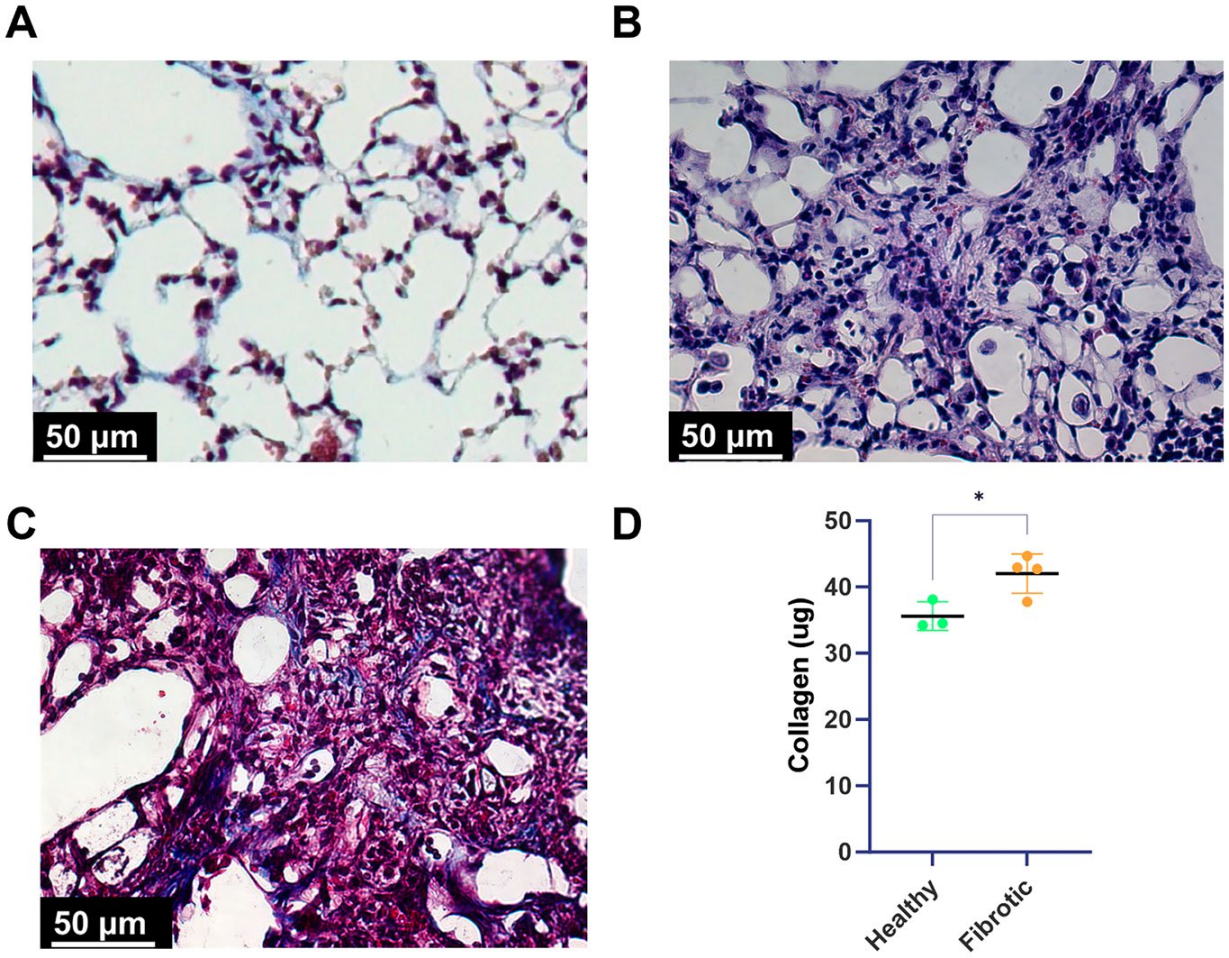
(A) Representative image of healthy mouse lung tissue stained for Masson's Trichrome. (B) Representative image of bleomycin treated fibrotic lung tissue stained for hematoxylin and eosin. (C) Representative image of Masson's Trichrome staining confirming the presence of abnormal alveoli structure and collagen staining. (D) PBS wash from the bleomycin treated explants contained significantly more collagen compared to the control as quantified by SirCol analysis. Healthy *N* = 3, Fibrotic *N* = 4. Statistics: Unpaired *t* test, *p < 0.05.

One healthy mouse and one mouse treated with bleomycin was used for immunohistochemical confirmation of fibrosis. After primary and secondary euthanasia was performed, a blunt syringe was inserted into a small slit in the trachea and the lungs were inflated with 1% low melting temperature agarose in PBS. The lungs were then isolated, cryoprotected in 30% sucrose in PBS, and cryoembedded in OCT (Tissue‐TEK). 10 μm thick cryosections were then taken and stained for hematoxylin & eosin and Masson's Trichrome to confirm the presence of fibrosis (Figure 1A–C). Following the isolation of the lungs, the PBS in which explants were stored was used for a SirCol Assay (BioColor) to quantify the amount of soluble collagen in the lungs of the bleomycin mice compared to the control mice (Figure 1D). This assay was conducted according to the manufacturer's instructions.

### 2.4 Synthesis of PEG‐RGDS, PEG‐PQ, and PEG‐Growth Factor Conjugates

Monoacrylate‐PEG‐succinimidyl valerate (PEG‐SVA, 3.4 kDa, Laysan Bio) was used to PEGylate peptides and growth factors purchased from GenScript as described previously [25]. In brief, the cell adhesion peptide RGDS and PEG‐SVA were dissolved in anhydrous dimethyl sulfoxide (DMSO, Sigma Aldrich) at a 1.1:1 M ratio (RGDS:PEG‐SVA). Diisopropylethylamine (DIPEA, Sigma Aldrich) was added as a proton scavenger at a molar ratio of 2:1 PEG‐SVA to DIPEA. The reaction proceeded under argon for 24 h under constant agitation. The PEG‐RGDS product was quenched with and dialyzed against ultrapure water using a 3.4 kDa molecular weight cut‐off (MWCO) regenerated cellulose ester dialysis membrane (SpectraPor, Repligen) with five water changes over 2 days, frozen to −80°C, and lyophilized for 48 h. Conjugation efficiency and purity were assessed via gel permeation chromatography (GPC; EcoSEC Elite, Tosoh Biosciences) with refractive index (RI), ultraviolet (UV), and multi‐angle light scattering (MALS) detectors.

The MMP‐2 and MMP‐9‐degradable sequence GGGPQG↓IWGQGK (PQ) was likewise reacted in anhydrous DMSO with DIPEA at a 2.1:2:1 M ratio to PEG‐SVA to form acrylate‐PEG‐PQ‐PEG‐acrylate (PEG‐PQ diacrylate) [13, 27]. The reaction mixture was blanketed with argon, allowed to react for 24 h, quenched, and purified, lyophilized, and characterized as described above. Proton NMR (Bruker Avance III 600 MHz, Billerica, MA) was used to confirm 99% acrylation of the diacrylate product.

PEG‐SVA was also coupled to the growth factors FGF‐2 and PDGF‐BB at molar ratios of 200:1 in sterile 50 mM sodium bicarbonate buffer (pH 8.5) for 4 days at 4°C followed by 4 h at 20°C to form acrylate‐PEG‐PDGF (PEG‐PDGF) and acrylate‐PEG‐FGF (PEG‐FGF). PEG‐growth factor conjugates (PEG‐GFs) were then lyophilized, resuspended in sterile HEPES‐buffered saline (HBS, pH 7.4), and conjugation was confirmed via silver ion precipitation (ProteoSilver Stain Kit, Sigma Aldrich) following polyacrylamide gel electrophoresis (SDS‐PAGE), as previously reported [18, 20, 25].

### 2.5 Formation of Hydrogels of Varying Stiffness

**FIGURE 2:**
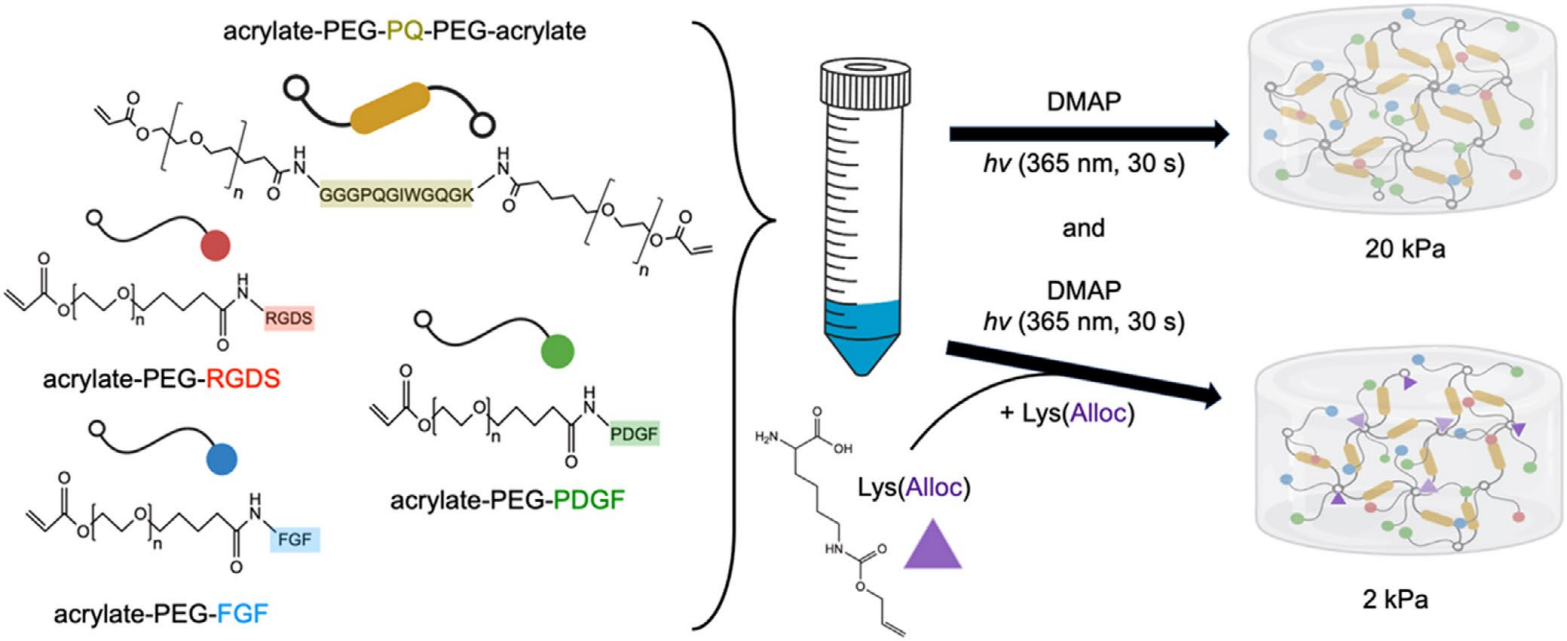
Hydrogels were fabricated from combinations of PEG‐peptide and ‐growth factor conjugates to model the extracellular microenvironment of healthy and fibrotic lung tissues.

Hydrogel precursors were formed by combining 3.5 mM PEG‐RGDS, 5% (w/v) PEG‐PQ, and 1% (v/v) photoinitiator solution (2,2‐dimethoxy‐2‐phenylacetophenone in N‐vinylpyrrolidone; DMAP, 300 mg/mL) in HBS. To reduce the stiffness of hydrogels independently of polymer density, *N*‐ε‐allyloxycarbonyl‐L‐lysine (Lys(alloc), Sigma Aldrich) was incorporated at a 3.5:1 M ratio of Lys(alloc):acrylate into the precursors to form gels with a compressive modulus of approximately 2 kPa, mimicking healthy lung tissue, while 5% PEG‐PQ alone resulted in a compressive modulus of 20 kPa, mimicking the stiff, fibrotic lung environment [1, 9]. Pre‐polymer solutions were passed through a 0.2 μm sterile syringe filter (Whatman Puradisc Regenerated Cellulose Syringe Filter, Cytiva) and sterile PEG‐GFs were then added to a final concentration of 0.7 nmol/mL PEG‐PDGF and 0.27 nmol/mL PEG‐FGF (Figure 2).

Mechanical characterization was performed by curing pre‐polymer solutions for 30 s under exposure to 365 nm UV light at 10 mW‐cm^−2^ in 8 mm (ID) polydimethylsiloxane (PDMS) molds between two SigmaCote‐treated glass coverslips to form flat hydrogels. Hydrogels were swollen in Dulbecco's phosphate‐buffered saline (DPBS, Gibco, Thermo Fisher Scientific) overnight and subject to a 5 μm‐s^−1^ compression rate until failure (Discovery Hybrid Rheometer, TA Instruments, New Castle, DE) [28]. Compressive modulus was taken as the slope of the linear region of the stress–strain curve immediately preceding mechanical failure, between 60% and 80% strain (*n* ≥ 3).

For ex vivo experiments, sterile precursors were cured for 30 s in 6 mm (ID) PDMS molds adhered to the bottom of glass well plates that were previously incubated with 2% (v/v) 3‐(trimethoxysilyl)propyl methacrylate (TMPSMA) in 95% EtOH (pH 4.5) for 4 days to methacrylate the glass surface. Hydrogels were rinsed in PBS overnight to remove unreacted polymer or photoinitiator and incubated in cell culture media until explant plating. Each resulting hydrogel was 1 mm thick with a diameter of 6 mm.

### 2.6 Explant Culture

For explant culture on hydrogel surfaces, one explant was placed atop each hydrogel in individual wells of a 24‐well plate. This allowed vessels to sprout both on the surface of the hydrogel and into the hydrogel while ensuring the tissue does not experience the stiffness of the underlying glass plate and preventing the need for the tissue to be exposed to UV light in the crosslinking process since the gel is fully formed. Each plate contained one row of 2 kPa hydrogels and one row of 20 kPa hydrogels and received only fibrotic explants or control explants. To minimize the effects of variable delivery of bleomycin, each row received two explants from each region of the lobe (upper, middle, and lower) for a total of six explants per group per plate. Of the 25–30 samples that were collected from each left lobe, six were plated per stiffness group. At the time of plating, 200 μL of Endothelial Growth Media 2 Microvascular formula (EGM‐2MV, Lonza) was added to each well. One hour after plating, an additional 100 μL was added, followed by a final addition of 200 μL 4 h later. This ensured the explants had proper time to adhere to the hydrogel and resulted in a final media quantity of 500 μL per well. Every other day, 250 μL of media was removed and saved for Luminex and ELISA analysis, and 250 μL of new media was added. Media was saved on Days 2, 4, 6, and 8, and saved media was stored at −20°C until time of use.

### 2.7 Brightfield Imaging

On Day 7, explants were imaged using the 10X objective to quantify sprout formation on a Leica Thunder Imager using a stage top incubator to maintain carbon dioxide and temperature levels during imaging. The edge of the explant was aligned with the edge of the field of view and a z‐stack was acquired to capture sprouts or cell infiltrate that entered the hydrogel. Each z‐stack was then combined using the extended depth of field tool in the LAS X software to produce a single compiled image for each field of view. This ensures that we are quantifying all vascular sprouts that occur in each group and not exclusively focusing on sprouts in one plane of view. Images were manually categorized as previously published [1] into images that contain discernable vascular sprouts (Figure 4E) versus images with disorganized infiltration of cells and potential vessels (Figure 4F). Vascular organization is a key part of vascular homeostasis and the formation of healthy vasculature. Images with discernable vascular sprouts were analyzed in ImageJ to quantify the number of sprouts per sample and length of sprouts in each sample.

### 2.8 MMP‐2 and MMP‐9 Analysis Using ELISA

At each media change (Days 2, 4, and 6) 250 μL of spent media was saved and frozen at −20°C. At the end of the experiment on Day 8 all media was saved. For each row in a 24‐well plate, the six wells were split evenly into three pooled samples (i.e., spent media from wells A1 and A2 were combined, wells A3 and A4 were combined, and so on). An MMP‐2 ELISA (R&D Systems, Minneapolis, MN) and MMP‐9 ELISA (R&D Systems, Minneapolis, MN) were run according to manufacturer's instructions and imaged on a plate reader at both 450 and 540 nm to quantify absorbance. Background signal was then subtracted out of the absorbance reading and the data was analyzed. To remain consistent with imaging measurements the data for each experimental group per plate was averaged to yield an *N* of 3 for healthy samples and 4 for fibrotic samples.

### 2.9 Luminex Angiogenesis Assay

Spent media from Day 6 of the time course was pooled using the same methods as were used for preparing the media for ELISA analysis. Then 20 μL of each sample was used to run a Luminex Mouse Angiogenesis Panel (Milliprix, Millipore Sigma) according to the manufacturer's instructions. To remain consistent with imaging measurements, the data for each experimental group per plate was averaged to yield an *N* of 3 for healthy samples and 4 for fibrotic samples.

### 2.10 Statistical Analysis

The left lung lobe of one mouse was used for each 24‐well plate and thus each mouse and therefore each plate was considered an independent sample, N. Each mouse had its left lung lobe dissected and cut into approximately 25 samples. One sample was plated per well for two rows of a 24‐well plate for where each row contained a different stiffness hydrogel. Each well was considered a technical replicate, *n*, with the set of six wells per stiffness in each plate being averaged to account for variability that is inherent to the assay. To avoid bias from variable bleomycin delivery to the entire lobe each treatment group received at least two technical replicates from the top, middle, and bottom of the left lung lobe that was harvested. All images acquired for each plate were averaged for each experimental subgroup. A random block design linear mixed model was used to evaluate the influence of each fixed affect (Treatment or Stiffness) and their interaction. Mouse number was considered a random effect in order to account for mouse‐to‐mouse variability. For the interaction model Sidak's post hoc test was used to identify differences between the estimated marginal mean of each subgroup which represents a prediction of the mean outcome adjusted for any influence from individual mice. Statistical significance in the individual fixed effect models (Treatment or Stiffness) is denoted with asterisks throughout the manuscript with *p < 0.05. Statistical significance within the interaction model is distinguished with a hashtag with ^#^ *p* < 0.05. All data are presented as average +/− standard deviation. All statistical analysis was performed in R Studio and visualized in GraphPad Prism.

## 3 Results

### 3.1 Mechanical Characterization

**FIGURE 3:**
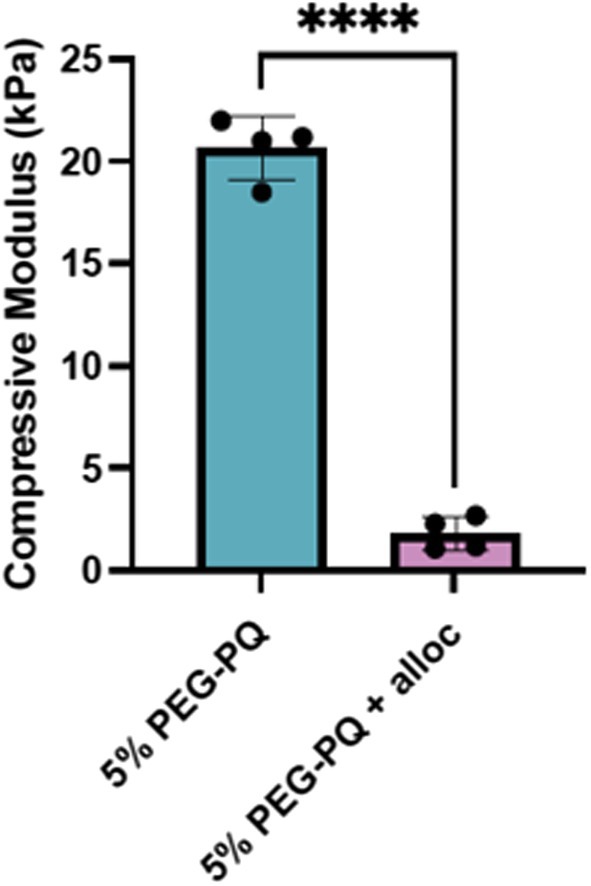
Compressive moduli of hydrogels fabricated from 5% (w/v) PEG‐PQ or 5% (w/v) PEG‐PQ with 3.5:1 alloc:Acrylate (**** = *p* < 0.0001).

Hydrogels of varying modulus were fabricated from precursors with constant polymer concentration of 5% with or without the addition of alloc groups at a 3.5:1 M ratio to acrylate end groups. Hydrogels (8 mm in diameter) were subjected to 5 μm‐s^−1^ compressive strain until failure and the compressive modulus was taken from the linear region of the slope of the stress–strain curve. Hydrogels fabricated from 5% (w/v) PEG‐PQ alone were shown to have a modulus of 20.6 ± 1.3 kPa; and the incorporation of Lys(alloc) at a 3.5:1 M ratio to PEG‐PQ acrylate end groups resulted in the fabrication of hydrogels of 1.8 ± 0.5 kPa (Figure 3).

### 3.2 Healthy and Diseased Lung Tissue Exhibited Differential Capillary Outgrowth Responses on Hydrogels of Varying Stiffness

Lung explants of mice treated with bleomycin to create fibrosis or littermate controls were cultured ex vivo on hydrogels mimicking healthy (2 kPa) or fibrotic (20 kPa) tissue stiffness for 7 days. During this time, microvessels emerged from the lung explants and grew into the hydrogels. Brightfield imaging was used to quantify four key metrics of microvessel outgrowth: (1) percent of samples with vessel sprouts, (2) average number of vessel sprouts per image, (3) average longest vessel length (μm), and (4) average shortest vessel length (μm). These measurements were made in ImageJ and quantify both overall vascularity (percent of samples with vessel sprouts) and the characteristics of the sprouts themselves.

**FIGURE 4:**
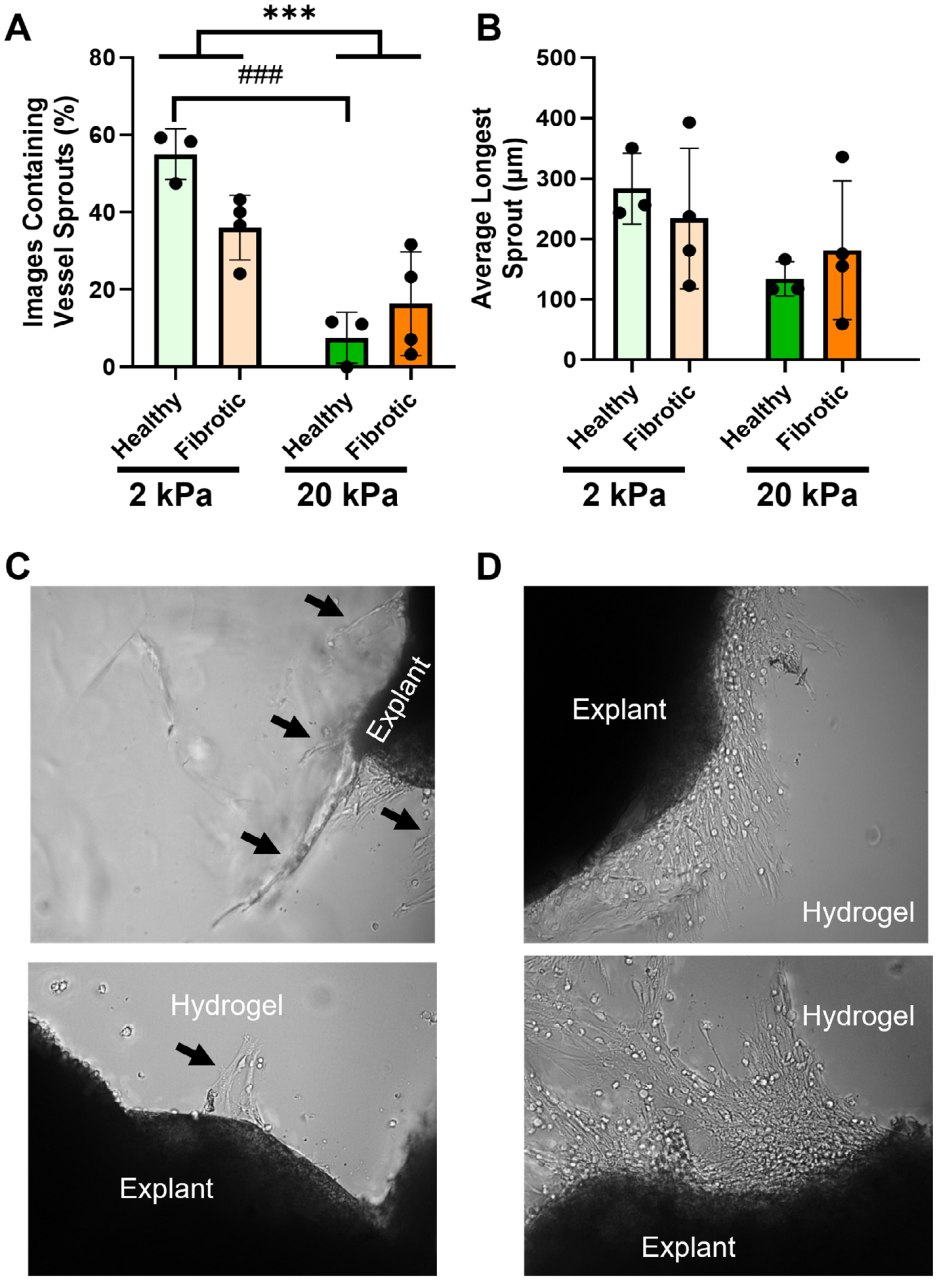
Metrics of microvascular outgrowth, as assessed via brightfield imaging at 10× magnification: (A) percent of samples containing vessel sprouts, (B) average longest sprout length (μm). (C) Representative image of well‐defined sprout from the control explant cultured on 2 kPa healthy lung explant group. (D) Representative image of the disorganized cell infiltration observed in the 20 kPa fibrotic lung explants. Organized vessel sprouts are denoted by a black arrow. Fibrotic mice *N* = 4, Healthy mice *N* = 3. Statistics: Random block design linear mixed model, Sidak's post hoc test evaluated differences between estimated marginal means, significant differences due to the effect of stiffness alone denoted by ***p < 0.001, significant differences due to the interaction of treatment and stiffness denoted by ^###^ *p* < 0.001.

Three random block design linear mixed models were developed for each output to assess the influence of (1) Bleomycin Treatment (Fibrosis), (2) Stiffness, or (3) the combined effect of Treatment of Stiffness on the output while removing mouse‐to‐mouse variability and the estimated marginal mean was compared for each. In the individual effect models it was observed that stiffness had a significant effect with the samples in 20 kPa hydrogels having an estimated mean of 7.9 (SEM ± 2.9) and the samples in 2 kPa having an estimated mean of 32.9 (SEM ± 4.9), however the individual model for treatment did not (Healthy: 7.7 SEM ± 4.3 vs. Fibrotic: 15.6 SEM ± 4.3). In the interaction model the healthy lung explants hydrogel stiffness significantly impacted the percent of samples that contained vessel sprouts (estimated means 43.2 SEM ± 8.0 vs. 5.7 SEM ± 4.4, actual means 55% ± 6.6% vs. 7.6% ± 6.6%), a finding which correlates with our prior study (Figure 4A) [1]. Contrastingly, the fibrotic lung explants were agnostic to hydrogel stiffness and the percent of samples containing vessel sprouts was not significantly different (estimated means 26.9 SEM ± 6.4 vs. 11.5 SEM ± 4.7, actual means 36% ± 8.4% vs. 16.4% ± 13.4%). Even though the stiffness was found to significantly impact the frequency at which samples contain vessels, it did not impact the characteristics of the vessels themselves, nor did the effect of treatment or the combined effect of stiffness and treatment. When we measured the average longest vessel in each sample we observed a trend where increased hydrogel stiffness hindered average longest sprout length in the healthy lung explants (2 kPa: 283.5 ± 58.5 μm vs. 20 kPa: 133.9 ± 28.3 μm) but this effect was not observed in the fibrotic lung explants (2 kPa: 233.8 ± 116.3 μm vs. 20 kPa: 219.5 ± 185.7 μm) and was not significant in either group (Figure 4B). These measurements were performed on well‐defined vessel sprouts as seen in the representative image in Figure 4C as opposed to disorganized sprouting infiltration where individual sprouts were different to identify (Figure 4D). Hydrogel stiffness did not significantly impact the average length of the shortest sprout or the average number of sprouts per sample in either healthy nor fibrotic lung explants (Figure S1).

### 3.3 Fibrotic Lung Explants Excrete Increased Angiogenic Cytokines

**FIGURE 5:**
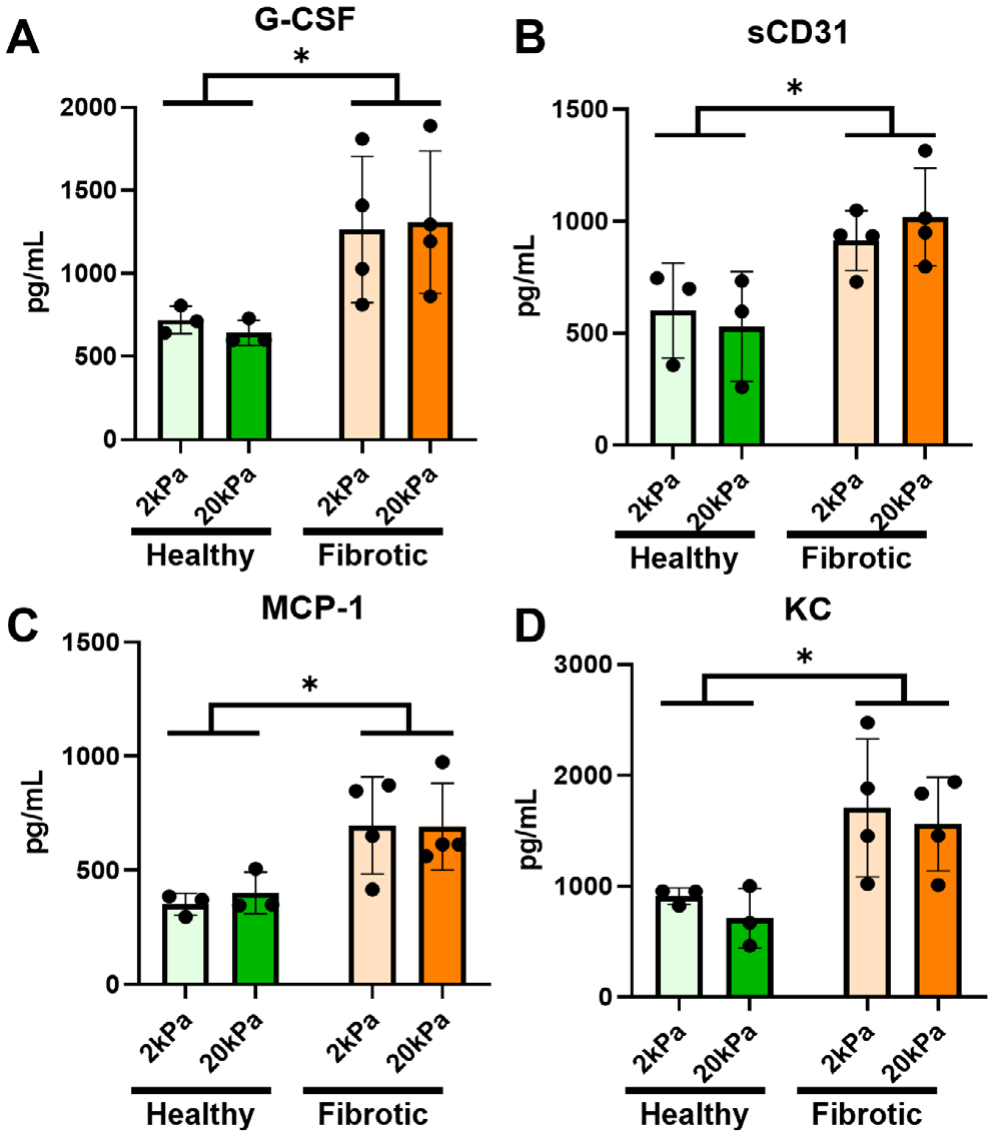
Luminex Angiogenesis Analysis reveals higher angiogenic and inflammatory cytokines in fibrotic samples on Day 6 compared to healthy controls (A) G‐CSF (B) sCD031 (C) MCP‐1 (D) KC Statistics: Healthy *N* = 3, Fibrotic *N* = 4, Random block design linear mixed model, Sidak's post hoc test evaluated differences between estimated marginal means, significant differences due to the effect of treatment with bleomycin (Healthy vs. Fibrotic) alone denoted by *p < 0.05.

To explore the underlying molecular differences that may be driving differences in sprout formation, we performed a Luminex assay on 27 analytes relevant to angiogenic signaling on supernatant preserved from Day 6. In our analysis, four analytes, granulocyte‐colony stimulating factor (G‐CSF) (healthy: 681.1 SEM ± 171.6 vs. fibrotic: 1286.7 SEM ± 148.6), monocyte chemoattractant protein 1 (MCP‐1) (healthy: 375.7 SEM ± 61.6 vs. fibrotic: 693.9 SEM ± 53.4), soluble CD‐31 (healthy: 565.2 SEM ± 84.1 vs. fibrotic: 966.1 SEM ± 72.8), and keratinocyte‐derived cytokine (KC) (healthy: 811.8 SEM ± 164.7 vs. fibrotic: 1634.9 SEM ± 142.6) were significantly higher in the fibrotic lung explants compared to the healthy lung explants in the treatment linear mixed model (Figure 5A–D). G‐CSF and MCP‐1 have been linked to matrix metalloprotease (MMP) expression, including inducing upregulation of MMP‐2 and MMP‐9 expression, which are the two MMPs capable of degrading the PEG‐PQ diacrylate backbone [29, 30, 31, 32, 33, 34].

### 3.4 Fibrotic Lung Explants Have Increased MMP‐2 Secretion, but Not MMP‐9 Secretion

**FIGURE 6:**
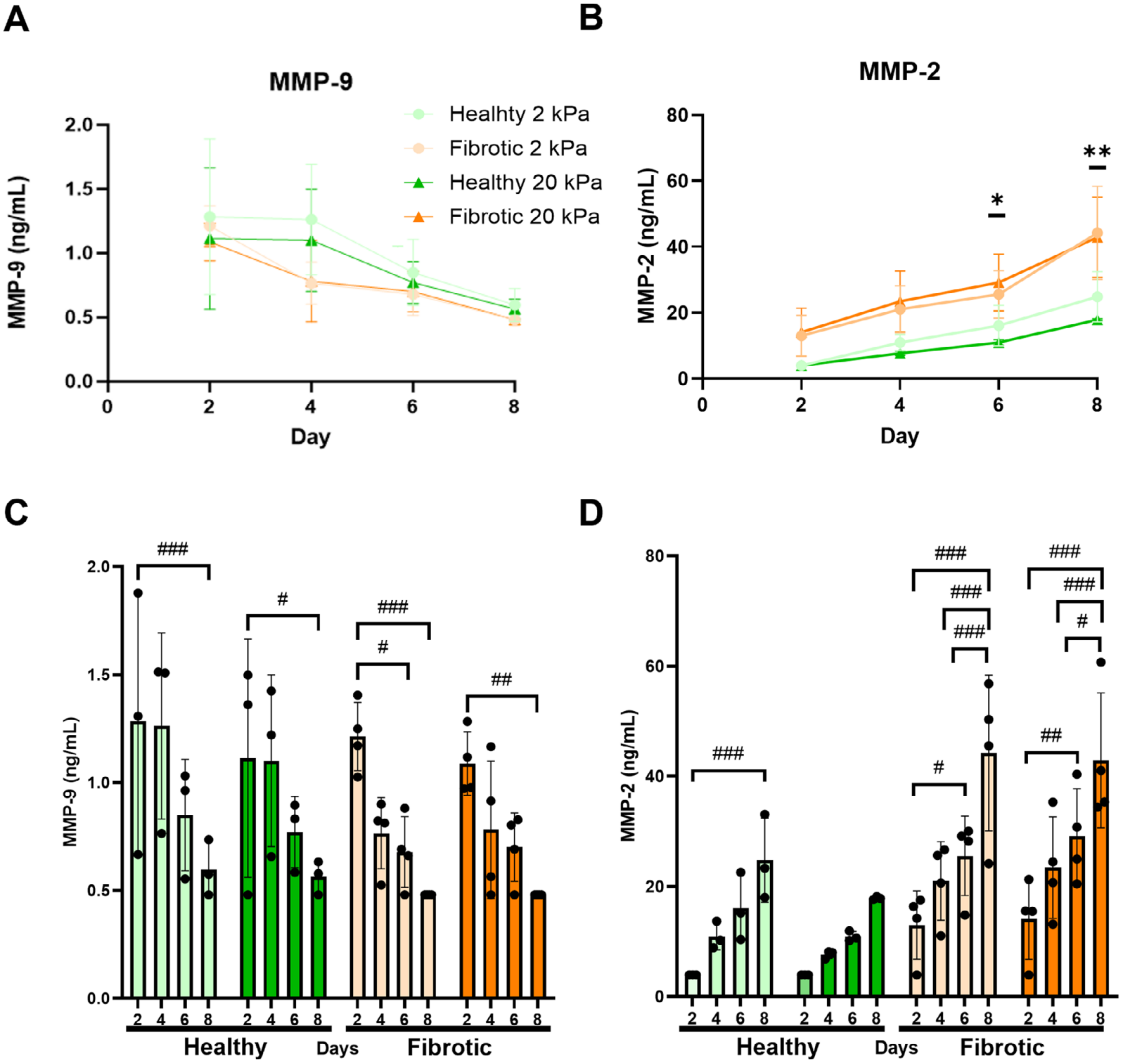
(A) MMP‐9 secretion decreases over the culture time course and is not significantly impacted by bleomycin induced fibrosis. (B) MMP‐2 increases over time and is significantly higher in the media from explants treated with bleomycin to induce fibrosis in later days in the time course. (C) When evaluating the combined effect of stiffness and treatment over time MMP‐9 decreases in all subgroups and is significantly lower on Day 8 in all groups compared to Day 2. (D) The increasing trend in MMP‐2 over time is observed in all groups. Statistics: Healthy *N* = 3, Fibrotic *N* = 4, Random block design linear mixed model, Sidak's post hoc test evaluated differences between estimated marginal means, significant differences due to the effect of treatment with bleomycin (Healthy vs. Fibrotic) alone denoted by *p < 0.05, **p < 0.01, significant differences due to the interaction of treatment and stiffness denoted by ^#^ *p* < 0.05, ^##^ *p* < 0.01, ^###^ *p* < 0.001.

MMP‐2 and MMP‐9 levels were analyzed in the supernatant of our samples at Days 2, 4, 6, and 8 using ELISA assays. MMP‐9 decreased over time in both groups, although the two groups were not significantly different at each time point (Figure 6A). In contrast, MMP‐2 increased over time and was markedly higher in the fibrotic lung explants, with the treatment model identifying significantly higher MMP‐9 in the fibrotic lung explants on Days 6 and 8 (Figure 6B). When we quantified the difference between the two treatment groups at each time point using a model of the interaction of stiffness and treatment, we found that MMP‐9 significantly decreased in all groups over time between Day 2 and 8 (Figure 6C). The increase in MMP‐2 over time was only significant in three of the four groups by Day 8. Interestingly, in the healthy 2 kPa tissues there is only a significant difference between Days 2 and 8, but in the fibrotic tissues Day 8 is significantly higher than all other time points for each stiffness group (Figure 6D).

## 4 Discussion

This application of mechanically tunable hydrogels demonstrates the ability of these materials to replicate different tissue environments in the context of disease. By examining the role of matrix stiffness and prior fibrotic injury on microvascular remodeling—specifically lung capillary sprouting—in the context of pulmonary fibrosis, we have demonstrated the significance of the mechanical environment and showcased the potential benefit of modular hydrogels as versatile platforms for elucidating complex disease mechanisms associated with changes in the mechanical characteristics of the extracellular matrix (ECM) while also being adaptable to different tissue sources. We found that stiffness significantly impacted the percent of samples containing vessel sprouts when considered in isolation, but the added interaction of treatment negated this effect in the fibrotic lung tissue, although it was still observed in the healthy lung tissue. This suggests that while hydrogel stiffness has an effect, it is limited when combined with prior injury and is more impactful on healthy tissue than tissue that already contains a stiff fibrotic injury. The effect of bleomycin injury to create fibrosis did have a significant impact on cytokine and MMP‐2 secretion when considered in isolation in a linear mixed model. Further, the interaction of fibrotic injury and stiffness significantly impacted both MMP‐9 and MMP‐2 secretion over time within each group, with MMP‐9 decreasing and MMP‐2 increasing over time. This suggests that over time, the explant was degrading the gel primarily through MMP‐2 secretion, as opposed to earlier time points where MMP‐9 was more prevalent. Overall, this study suggests that damaged lung is primed to withstand stiff environments in a way that supports sprouting, possibly through the upregulation of MMP‐2.

Bleomycin treated lungs are known to have an initial strong inflammatory response that wanes over time. It is well accepted that the inflammatory stage of the bleomycin induced lung damage has mostly resolved by Day 14 and that by Day 28, which is our time point, the only prolonged effect of the bleomycin treatment is the fibrosis. The bleomycin mouse model is currently considered the standard model in the field with timepoints between Days 14 and 28 being appropriate for assessing fibrotic damage without confounding inflammation [23, 24, 25, 26]. Therefore, the elevated levels of cytokines and MMPs observed in this study are not a residual of the initial bleomycin injury, but perhaps suggest a sensitivity to new stimuli after an initial injury. For example, a previous study analyzed MCP‐1 expression post bleomycin administration in rats and found that while it increased in the early phase (Days 3–7) by Day 21 it had decreased back to levels similar to the control mice [35]. This suggests that the elevated levels observed in our system are not due to the initial administration but perhaps a sensitivity to new stimuli that results in an overreaction of cytokine secretion after a prior injury. These elevated levels of inflammatory cytokines align with the in vivo study observations and suggest the fibrotic lungs retain some memory of the initial injury, affecting their ability to respond to the hydrogel environment.

More work is needed to fully elucidate the mechanisms mediating the ability of fibrotic lungs to sprout in stiffer environments. For example, we observe opposing trends over time of MMP‐2 and MMP‐9 secretion suggesting a shift in signaling toward an MMP‐2 mediated sprouting once the culture is well established. Future approaches may include inhibition of MMP‐2 or other angiogenic factors to explore this further and determine the specific role of MMP‐2 in driving this sprouting phenotype. Additionally, this work sets a precedent for using hydrogel‐based models to study other fibrotic diseases, such as kidney and tumor fibrosis. The ability to tailor hydrogel properties to mimic different stages of fibrosis could enable a better understanding of tissue remodeling in these states and the development of more accurate models or targeted interventions.

Despite the advantages of our synthetic hydrogel platform, other features of the native ECM, such as biochemical gradients of soluble factors or structural proteins, are notably absent, which addresses the influence of microenvironmental stiffness in a reductive, angiogenic setting. Additionally, while we were able to show that matrix stiffness had a negative impact on angiogenic sprouting in healthy tissue, other mechanical properties of the ECM that are affected by fibrosis, such as viscoelasticity or molecular topography, may also play a role in the dysregulation of capillary sprouting. Another limitation of our model is that for the secreted factors we cannot isolate those that are secreted exclusively by the sprouts from those that are secreted by the explant, thus limiting our interpretation of the data. The dynamic changes in MMP secretion over time suggests a response to the hydrogel environment but cannot be confirmed. Additionally, we used carbon dioxide asphyxiation as the sacrifice method for the mice and there are other potential methods which may prove more effective, such as exsanguination via vessel flushing to preserve fresh lung tissue. Lastly, while the bleomycin treated mouse model is the standard murine model for studying fibrosis there are inherent limitations to the model including the potential for variable delivery of and response to bleomycin, and regional heterogeneity. While we address this issue to the best of our ability by sampling from different lung regions and using multiple mice for our explants, future studies could include use of other fibrotic lung models such as TGF‐β overexpression, silicosis, or repeat‐dose bleomycin [36, 37].

## 5 Conclusions

Our PEGDA‐based hydrogel model reveals critical differences in the angiogenic potential of diseased and healthy lung tissue cultured on substrates that mimic healthy and fibrotic extracellular conditions. Notably, we observed distinct differential responses in both microvessel sprouting and secreted factors (inclusive of growth factors, MMPs), which together suggest adaptation of fibrotic lung tissue to enhanced mechanical stiffness. Interrogation of specific cell‐matrix interactions that drive these responses, such as ligand‐initiated intracellular signaling or cytoskeletal dynamics, may further clarify the role of matrix mechanics in disease. The modular nature of our hydrogel model provides a versatile platform for the dissection of various intracellular and substrate‐mediated pathways involved in tissue remodeling in diverse disease states. Adaptation of the versatile angiogenic hydrogel platform may contribute to the development of models for interrogation of similar stiffness‐dependent mechanisms associated with other organ or tissue systems in diverse pathological environments.

## 6 Perspective

1. Built upon prior work that utilizes ex vivo hydrogel based sprouting assays by incorporating previously injured tissue. Tissue from fibrotic mice had an enhanced response to a stiff hydrogel environment compared to explants from healthy mice.
2. This enhanced response included higher levels of angiogenic cytokines and MMPs, especially MMP‐2 which is one of the MMPs that can degrade the hydrogel.
3. Future studies should interrogate these secreted cues further to understand how prior fibrotic injury affects the microvascular remodeling response

## Author Contributions

Figure and manuscript preparation: Julie Leonard‐Duke, Samuel M. J. Agro, Riley T. Hannan, Shayn M. Peirce, and Lakeshia J. Taite. Conceptualization: Julie Leonard‐Duke, Riley T. Hannan, Lakeshia J. Taite, and Shayn M. Peirce. Study design: Julie Leonard‐Duke, Riley T. Hannan, Jeffrey M. Sturek, Lakeshia J. Taite, and Shayn M. Peirce. Data collection: Julie Leonard‐Duke, Samuel M. J. Agro, David J. Csordas, Riley T. Hannan, and Lakeshia J. Taite. Data analysis: Julie Leonard‐Duke, Samuel M. J. Agro, David J. Csordas, and Riley T. Hannan. Analysis and interpretation of results: Julie Leonard‐Duke, Samuel M. J. Agro, Lakeshia J. Taite, and Shayn M. Peirce. The manuscript was written through contributions of all authors. All authors have given approval to the final version of the manuscript.

## Supporting information


Figure S1.

## Data Availability

Data available upon request from corresponding author.
