# Supplementary material for: Mechanically Tunable Poly(Ethylene Glycol) Diacrylate Hydrogels Reveal Stiffness‐Related Impairments in Capillary Sprouting in Experimental Lung Fibrosis
Source: Microcirculation. 2025 Jul 17;32(5):e70018. doi: 10.1111/micc.70018 (PMC12271678; doi:10.1111/micc.70018)
Supplement: Supplementary file 1 — Figure S1. [file MICC-32-e70018-s001.docx]

**Supplementary Material**

Title: Mechanically tunable poly(ethylene glycol) diacrylate hydrogels reveal stiffness-related impairments in capillary sprouting in experimental lung fibrosis

| **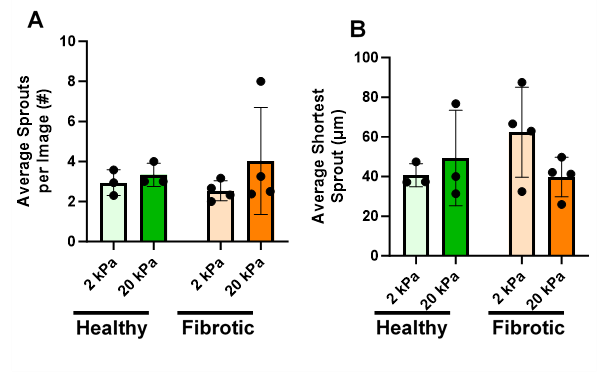** |
| --- |
| **Supplementary Figure 1**. Metrics of microvascular outgrowth, as assessed via brightfield imaging at 10X magnification: A) average number of sprouts per image, B) average shortest sprout length (µm). Fibrotic mice N = 4, Control mice N = 3. Statistics: Random block design linear mixed model, Sidak’s post-hoc test evaluated differences between estimated marginal means. |
